# Drought-driven shifts in *Eucommia ulmoides* rhizosphere mycobiota and metabolites mediate host tolerance

**DOI:** 10.1128/spectrum.00847-25

**Published:** 2025-07-11

**Authors:** Chenglong Li, Shuangshuang Hou, Jinling Li, Xueqian Zhang, Qingsong Ran, Yanfeng Han, Zhijie Wang, Chunbo Dong

**Affiliations:** 1 Department of Ecology/Key laboratory of Plant Resource Conservation and Germplasm Innovation in Mountainous Region (Ministry of Education), College of Life Sciences, Institute of Fungus Resources, Guizhou University 71206, Guiyang, Guizhou, China; 2 Guizhou Key Laboratory of Agricultural Microbiology, Guiyang, Guizhou, China; Guangdong Academy of Sciences, Guangzhou, Guangdong, China

**Keywords:** medicinal plants, fungal diversity, core microorganisms, plant physiology, root metabolites

## Abstract

Drought poses considerable challenges to the sustainable development of crops, highlighting the urgent need to improve plant resistance to drought stress. Rhizosphere mycobiota roles in *Eucommia ulmoides* drought adaptation remain uncharacterized. This study examines *E. ulmoides’* rhizosphere mycobiota diversity, its changes, and interactions with plant physiology and metabolites under drought stress using amplicon sequencing, plant physiological assessments, and non-targeted root metabolomics. Our data indicate that drought stress considerably altered the species richness and community composition of the *E. ulmoides* seedling rhizosphere mycobiota, affecting the co-occurrence patterns and the composition of core fungal taxa within the mycobiota. Additionally, *Sordariomycetes* were notably enriched in the rhizosphere of *E. ulmoides* under drought stress and showed a notable positive correlation with the physiological indicator soluble sugar (SS). During drought stress mid-stages, rhizosphere core fungal taxa of *E. ulmoides* exhibit higher diversity, increased network connectivity, and a tighter network structure. Correlation analyses show that core fungal taxa are significantly linked to malondialdehyde (MDA) content. The root metabolome’s phosphatidylcholines (o-16:0/22:6(4Z,7Z,10Z,13Z,16Z,19Z)) and 8-demethyltetracenomycin C are also notably affected by the core microbial taxa. In summary, drought stress drives changes in the *E. ulmoides* rhizosphere mycobiota, plant physiology, and root metabolites, with MDA, SS, and 8-demethyltetracenomycin C possibly mediating the selection of specific rhizosphere fungal communities. Taken together, these data provide notable insights into plant-microbe interactions under drought stress and have important implications for improving the drought adaptability of *E. ulmoides*.

**IMPORTANCE:**

Drought presents substantial challenges to the sustainability of crops, highlighting the need to enhance their resistance to arid conditions. Although the rhizosphere microbiome plays a crucial role in bolstering crop resilience, the dynamics and mechanisms of *Eucommia ulmoides*‘ rhizosphere mycobiota under drought conditions remain poorly understood. This study provides valuable insights into the interactions between plants and microbes under drought stress and has significant implications for improving the drought adaptability of *E. ulmoides*.

## INTRODUCTION

Global climate change, with its increasing frequency of drought events, has become one of the most destructive environmental stresses in crop production (1). Drought can affect plant water potential and swelling, disrupting plant functions and altering physiological and morphological characteristics (2). The root system is the primary organ through which plants sense soil moisture deficits, conveying various signals to the plant under different drought conditions (3). Moreover, plant roots engage in complex interactions with soil microbial communities, co-shaping unique rhizosphere microbial communities. These microbial communities are not only crucial for plant growth and health but also exhibit sensitive responses to environmental changes, adapting to various environmental conditions through their biological functions (4).

As a microecological zone that connects plant roots to the soil, the rhizosphere contains a multitude of soil microbial communities that participate in complex ecological and biological processes (5). Existing research indicates that the rhizosphere microbiome is closely related to host development (6, 7), nutrient absorption (8), and pathogen immunity (9). Furthermore, the role of the rhizosphere microbiome in mitigating abiotic stress in crops has recently garnered increased attention (10, 11). Relevant studies have shown that under drought stress, plant-driven rhizosphere microbial communities undergo changes to improve drought resistance and growth development under arid conditions (12). Specifically, drought alters the nutrient availability and living environment of the rhizosphere microbial community, thereby affecting their diversity, composition, and stability (13, 14). However, despite the fact that fungi may exceed bacteria in biomass within terrestrial ecosystems (15), research into plant-associated fungal communities is relatively lacking compared to bacteria (16). Importantly, recent studies have shown that soil fungi in the plant rhizosphere have significant potential to alleviate plant drought stress (10, 17). For instance, research by Pan et al. has shown that plants can induce structural reorganization of the rhizosphere mycobiota. When faced with environmental challenges such as drought, these restructured fungal communities can significantly enhance a plant’s capacity to adapt to adversity. Interestingly, the authors of that study also revealed that in soil environments adapted to drought stress, generalist fungi with broad ecological niches show a more pronounced advantage compared to specialist fungi with narrow ecological niches (18). A study by Ferus et al. demonstrated that *Beauveria bassiana*, primarily recognized as an entomopathogenic fungus, helps seedlings of *Quercus rubra* L. to cope with drought stress (19). Additionally, previous studies have shown that arbuscular mycorrhizal fungi (AMF) improve plant resistance to drought. For example, Tang et al. found that AMF have a significant potential to mitigate the adverse effects of drought on plant growth. Taken together, there is a considerable body of evidence to suggest that fungi support plant growth and survival under drought conditions by enhancing physiological functions, including promoting biomass accumulation, improving the efficiency of photosynthesis, and increasing the absorption capacity for key resources such as nutrients and water (20).

Similarly, certain root exudates, which act as metabolic products of stress resistance, can alter the physical and chemical properties of the soil (21, 22). These compounds seep from the plant roots into the rhizosphere, mediating the transformation of the rhizosphere soil microbiome, thereby determining nutrient cycling and metabolism within the rhizosphere and playing a key role in influencing host plant drought adaptability (23, 24). Previous studies have shown that the chemical composition of root exudates has a direct impact on the rhizosphere community, thereby improving drought resistance. For instance, the fumaric acid seeping from banana roots can attract the growth-promoting rhizobacterium, *Bacillus subtilis* N11, to stimulate biofilm formation (25). Moreover, malic acid released by *Arabidopsis* can stimulate binding of *B. subtilis* strain FB17 to the roots, facilitating biofilm formation on the root surface (26). Additionally, studies have indicated that physiological indicators, such as proline (Pro) content, superoxide dismutase (SOD) activity, and malondialdehyde (MDA) content, in leaves can, to a certain extent, reflect the degree of drought stress experienced by plants (27).

*Eucommia ulmoides* Oliver, a tree species that has survived since the third glacial period, possesses significant medicinal and economic value (28). *E. ulmoides* also has a wide growth range and strong adaptability in China, primarily distributed in regions such as Hunan, Guizhou, and Sichuan. However, the southwestern region of China is home to one of the largest continuous karst landscapes in the world, covering an area of approximately 550,000 km^2^. The karst ecosystem is characterized by scarce soil, shallow soil layers, high permeability, and a predisposition to drought and water scarcity (29, 30). As a result, plants growing in karst regions are often subject to drought stress (31). For *E. ulmoides*, which is widely distributed in karst regions, drought stress is a key environmental pressure limiting its growth and yield. However, changes in the rhizosphere mycobiota of *E. ulmoides* under drought stress and their impact on plant drought resistance are currently not well understood. Given this context, we used a pot experiment to simulate the drought conditions of the karst region. Through amplicon sequencing, plant physiological indicators, and non-targeted root metabolomics, we sought to answer the following scientific questions: (i) is the drought adaptability of *E. ulmoides* in the karst landscape related to the structure and composition of its rhizosphere mycobiota? (ii) do the core fungi in the *E. ulmoides* rhizosphere undergo changes during plant drought adaptation processes? (iii) under drought conditions, are the rhizosphere metabolites and plant physiological traits associated with changes in the fungi?

## MATERIALS AND METHODS

### Experimental materials and soil

Seeds of the “Hua Zhong No. 8” *E. ulmoides* variety were used as the experimental material. The experimental soil was collected from the Guizhou University Farm, located in Guiyang, Guizhou Province, China (latitude 26°3*'*N, longitude 106°4*'*E), which is situated in a typical karst region with unique geological characteristics of karst soil. The collected soil was processed to remove plant debris, roots, gravel, and other large particulate impurities. The soil was then thoroughly mixed and evenly distributed into plastic pots, with approximately 2 kg of soil per pot. The specifications of the pots are as follows: height 16.5 cm, base diameter 12.4 cm, and mouth diameter 15.7 cm. The environmental factors, such as pH, nitrogen, phosphorus, and potassium, were uniform across all groups; basic nutritional components of the soil are presented in Table S1.

### Cultivation and drought treatment of *E. ulmoides* seedlings

*E. ulmoides* seeds were peeled and soaked in sterile water for 12 hours, followed by disinfection with 75% (vol/vol) alcohol for 2 minutes, before being rinsed three to four times with sterile water. The surface-disinfected seeds were placed in a culture box for germination under laboratory conditions with temperatures maintained between 22°C and 25°C, a light intensity of 2,500 lx, a photoperiod of 12–14 hours daily, and constantly maintained moisture. After 5 days of cultivation, seeds with uniform germination were sown in the aforementioned plastic pots, with three seeds per pot. One week after germination, seedlings with inconsistent growth were removed. The pots were then placed in a natural environment for 120 days, after which seedlings with uniform growth were selected and moved back to the cultivation chamber for continued cultivation. The cultivation chamber was controlled at a temperature of 20°C–25°C. Sampling began 24 hours after the last watering, with the first sample taken, followed by sampling every 2 days thereafter, until the sixth sampling when the *E. ulmoides* seedlings showed permanent wilting and sampling was terminated. The soil moisture levels at the time of the six samplings are detailed in Table S2. Including the control treatment, the experiment consisted of a total of six treatments, with six biological replicates per treatment. The experimental design is presented in Fig. S1.

### Soil sample collection

The entire root system of the *E. ulmoides* seedling was carefully extracted from the plastic pots. Subsequently, while wearing sterile gloves, the roots were gently shaken and rubbed to remove larger soil clumps and then placed into a 50 mL sterile tube containing 20 mL of sterile, pre-cooled 10 mM phosphate-buffered saline (PBS) solution (130 mM NaCl, 7 mM Na_2_HPO_4_, 3 mM NaH_2_PO_4_, pH 7.4). Next, the samples were centrifuged at 10,000 rpm for 15 minutes to form fine sediment particles, defined as the rhizosphere soil. Ultimately, the rhizosphere soil was rapidly frozen in liquid nitrogen and stored at −80°C for subsequent high-throughput sequencing.

### DNA extraction, PCR amplification, and high-throughput sequencing

Rhizosphere soil DNA was extracted using the cetyltrimethylammonium bromide (CTAB) method. The detailed methods and steps are provided in the Supplemental File. Internal transcribed spacer (ITS) sequences of fungal DNA from rhizosphere soil were amplified by PCR using specific primers designed for fungal ITS (forward primer [F]: 5′-CTTGGTCATTTAGAGGAAGTAA-3′; reverse primer [R]: 5′-GCTGCGTTCTTCATCGATGC-3′). The PCR reaction mixture (20 µL) consisted of 4 µL of 5× PCR buffer, 2 µL of 2.5 mmol/L dNTPs mix, 0.8 µL of each forward and reverse primer (5 µmol/L), 0.4 µL of rTaq DNA polymerase, 0.2 µL of bovine serum albumin, and 10 ng of template DNA. The reaction mixture was brought up to the final volume with nuclease-free water (ddH_2_O). The PCR amplification conditions were as follows: an initial denaturation step at 95°C for 3 minutes, followed by 35 cycles of denaturation at 95°C for 30 seconds, annealing at 55°C for 30 seconds, extension at 72°C for 45 seconds, final extension step at 72°C for 10 minutes, and then the samples were held at 10°C until the PCR cycle was complete. Excess primers, dNTPs, and enzymes were removed using a PCR purification kit, and the purified PCR products were used to construct sequencing libraries.

The prepared libraries were sequenced using the Illumina NovaSeq 6000 platform (Illumina Inc., San Diego, California, USA) using the associated NovaSeq 6000 S4 Reagent Kit (Illumina Inc., San Diego, California, USA). The resulting sequencing data were then uploaded to the National Center for Biotechnology Information (NCBI). The high-throughput Sequencing data were uploaded to the (NCBI database (BioProject ID: PRJNA1151941).

### Determination of plant physiological indices

To determine plant physiological indicators, 2 g of mixed leaf samples was placed into a plastic centrifuge tube containing 18 mL of pre-cooled 10 mM PBS solution. The leaf tissues were homogenized using a blender. Following centrifugation at 4°C for 20 minutes at 3,000 rpm, the supernatant was collected and stored at 4°C for subsequent analysis of various physiological indicators. We measured ascorbic acid (AsA), glutathione oxidized (GSSG), malondialdehyde, proline, superoxide dismutase, and soluble sugar (SS) content. All measurements were performed using a plant enzyme-linked immunosorbent assay kit (Mibio, Shanghai, China) according to the manufacturer’s instructions.

### Root collection and metabolomic sequencing

After removing the entire root system and rinsing with running water to eliminate surface soil and other impurities, fine roots were selected using tweezers and soaked in 70% (vol/vol) ethanol for 30 seconds, followed by rinsing with sterile water. The roots were then treated with sodium hypochlorite solution for 3 minutes before being rinsed again with sterile water. This process was repeated three to four times, and the roots were then blotted dry with sterile paper towels. Subsequently, the root samples were lyophilized using liquid nitrogen, homogenized using a sterilized mortar and pestle, and transferred to 2 mL Lysing Matrix E tubes before storage at −80°C until later use.

To prepare the root samples, 50 mg of root material was added to 1 mL of extraction solution containing 20 mg/L of internal standard (a mixture of methanol, acetonitrile, and water in a volumetric ratio of 2:2:1) and mixed for 30 seconds in a vortex mixer. Steel beads were then added, and the mixture was processed with a grinding apparatus at 45 Hz for 10 minutes, followed by ultrasonication in an ice-water bath for 10 minutes. The mixture was allowed to stand at −20°C for 1 hour before centrifugation at 4°C for 15 minutes at 12,000 rpm. Next, 500 µL of the supernatant was carefully transferred to a fresh Eppendorf tube, and the extract was dried in a vacuum concentrator. The dried metabolites were reconstituted by mixing with 160 µL of extraction solution (a 1:1 mixture of acetonitrile and water), vortexed for 30 seconds, and then subjected to ultrasonication in an ice-water bath for 10 minutes. After centrifugation at 4°C for 15 minutes at 12,000 rpm, 120 µL of the supernatant was carefully transferred to a 2 mL injection vial, and 10 µL of the mixture was taken for each sample to create a quality control sample for subsequent instrument analysis.

### Co-occurring networks and the definition of core microorganisms

Based on the Spearman correlation coefficient, we conducted co-occurrence network analyses on the microbial community data using the psych and WGCNA packages in R software (version 4.4.0). To mitigate the compositional bias in the microbial community data, we filtered for fungal taxa (at the genus level) present in more than 80% of the samples and retained only those correlations that were strong and statistically significant (Spearman’s |*r*| >0.6, p < 0.05) for the network analyses. Subsequently, we utilized Gephi software (version 0.10) for network visualization and the computation of topological parameters. We identified the core microbial taxa in the rhizosphere of *E. ulmoides* at various stages of drought based on network topological parameters, including degree (the number of direct connections of a node) and closeness centrality (a measure of how close a node is to all other nodes in the network). Specifically, taxa with high values in both degree and closeness centrality were defined as the core microbial taxa in the rhizosphere of *E. ulmoides*.

### Data analysis and visualization

To mitigate compositional biases in microbial community data, we filtered fungal taxa (at the genus level) present in the samples, retaining only those units that were present in more than 80% of the samples and exhibited strong correlations with statistical significance (Spearman’s |*r*| >0.6, *P* < 0.05) for subsequent data analysis. Data processing and statistical analyses were performed using Microsoft Excel 2021, SPSS (version 22.0), and R (version 4.4.0). Visualization analysis was performed on the OmicStudio bioinformatics online platform (https://www.omicstudio.cn/) and the Tutu cloud platform (http://cloudtutu.com.cn/).

## RESULTS

### Drought affects the diversity and community composition of the *E. ulmoides* rhizosphere mycobiota

**Fig 1:**
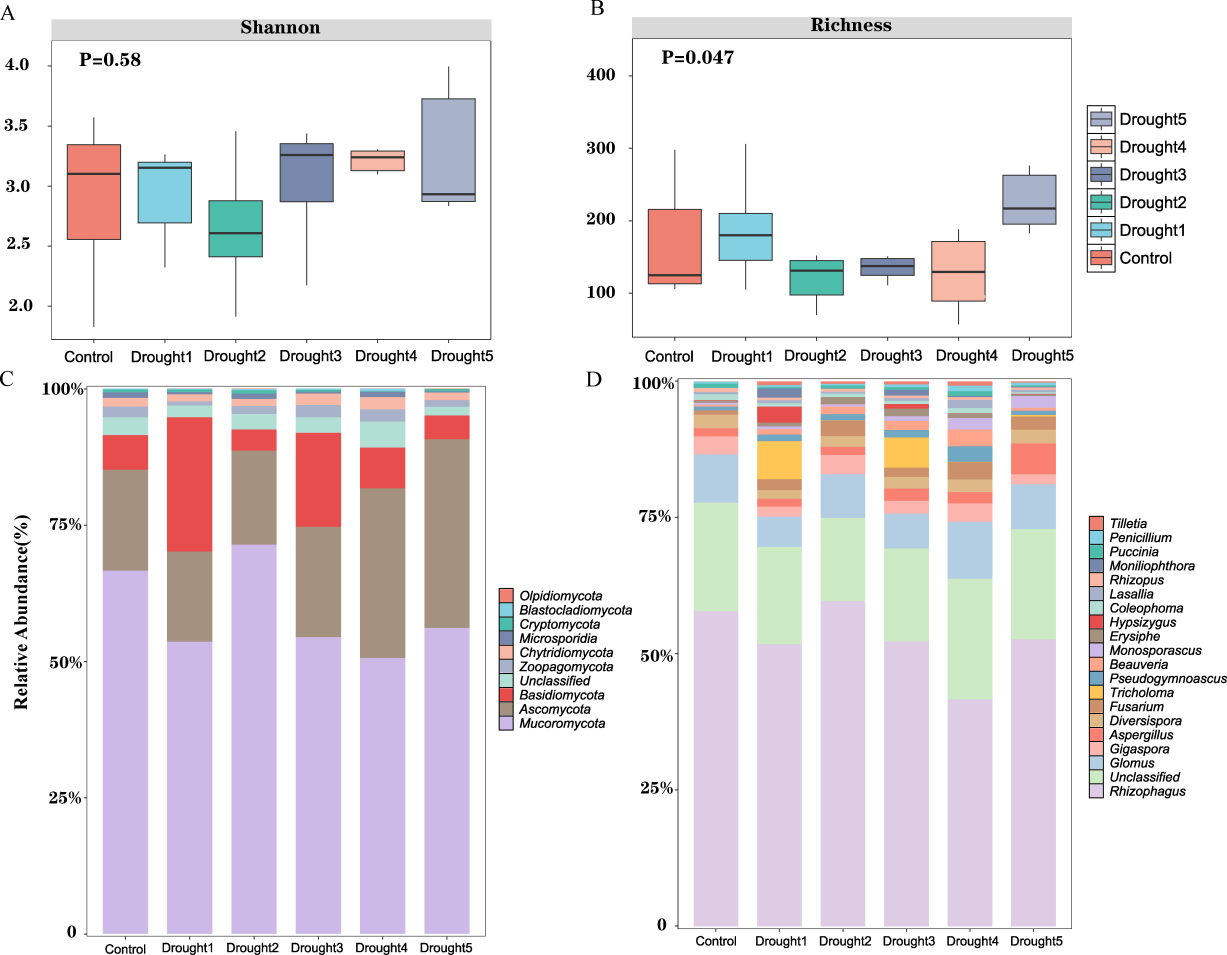
(**A and B**) Characteristics of the alpha diversity of rhizosphere microbes under drought stress in *E. ulmoides*. Composition and relative abundance of the top 20 (C) phyla and (D) genera.

In this study, we found that the diversity index of the community (i.e., the Shannon index) did not show significant differences between the control group and the various treatment groups (*P* > 0.05) (Fig. 1A). However, a significant difference in species richness was found between the control group and the drought treatment groups (*P* < 0.05), especially in the later stages of drought treatment, where the drought 5 treatment group exhibited the highest species richness (Fig. 1B). This phenomenon indicates that drought stress may have a significant impact on the species richness of the rhizosphere mycobiota.

At the species composition level, the Mucoromycota phylum was dominant in both the control group and all treatment groups, with its relative abundance significantly higher in the drought 2 treatment group compared to other groups. Additionally, the Basidiomycota phylum showed a significantly higher relative abundance in drought 1 and drought 3 treatment groups compared to the other control and treatment groups. Most notably, the Ascomycota phylum exhibited a significantly higher relative abundance in the Drought 4 and Drought 5 treatment groups (Fig. 1C; middle and late stages of drought treatment), suggesting that *Ascomycota* may play a crucial role in responding to drought stress.

At the genus level, the dominant genus in both the control and treatment groups was the AMF, *Rhizophagus*, with its relative abundance fluctuating across different groups but generally remaining highly abundant (Fig. 1D). Importantly, the relative abundance of *Tricholoma* was significantly higher in the drought 1 (6.99%) and drought 3 (5.54%) treatment groups compared to the control (0.01%) and drought 5 (0.2%) groups. Moreover, *Tricholoma* was not detected in the drought 2 and drought 4 treatment groups. Taken together, we can conclude that drought stress significantly altered the species richness and community composition of the *E. ulmoides* seedling rhizosphere microbiome, thereby affecting the relative abundance of different microbial taxa.

### Sordariomycetes fungi are significantly enriched in the rhizosphere of *E. ulmoides* under drought stress

**Fig 2:**
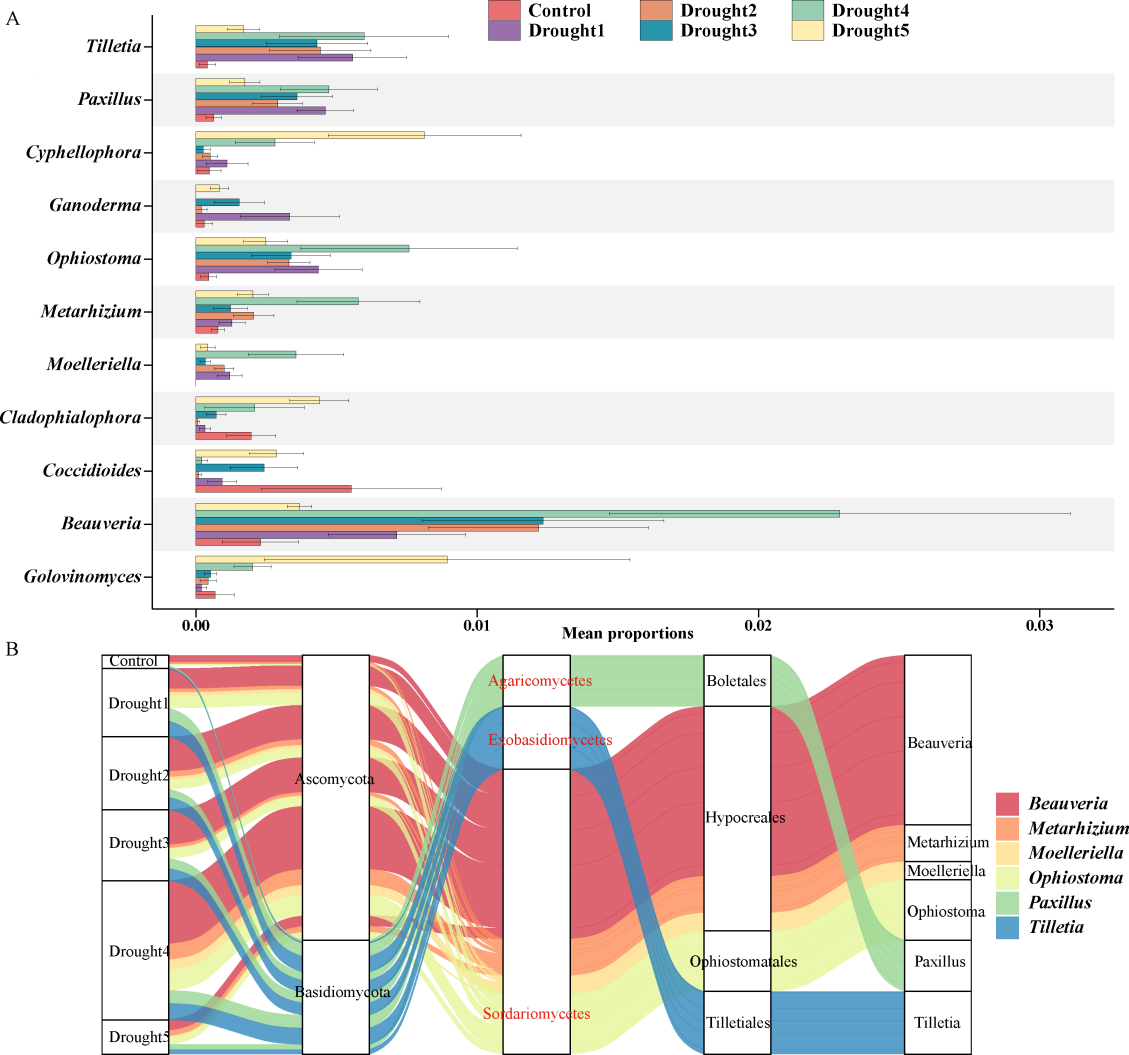
Characteristics of changes in *E. ulmoides* rhizosphere microbes under drought stress. (**A**) Stamp analysis of taxonomic taxa at the genus level based on Kruskal.test statistics. (**B**) Phylogenetic relationships of enriched microbial taxa based on taxonomic information annotated by amplicon sequencing.

Differences between the dominant genera across different treatment groups were analyzed using the Kruskal-Wallis test (Kruskal.test) (Fig. 2A). The results showed that, compared to the control group, the relative abundance of genera such as *Beauveria, Ophiostoma, Tilletia, Paxillus, Metarhizium,* and *Moelleriella* significantly increased in the treatment groups, and their relative abundance exhibited a trend of initially increasing and then decreasing with the increase in drought treatment intensity. Notably, in the drought 4 treatment group, the relative abundance of *Ophiostoma, Metarhizium, Moelleriella*, and *Beauveria* all reached peaks, with each being more than seven times that of the control group. Similarly, in the drought 5 treatment group, the relative abundance of *Cyphellophora, Cladophialophora,* and *Golovinomyces* also showed a significant increase compared to the control and other treatment groups. However, the genus *Coccidioides* had the highest relative abundance in the control group and showed a trend of increasing first and then decreasing with the increase in drought treatment intensity. These observations reveal the significant impact of drought stress on the structure of the rhizosphere mycobiota of *E. ulmoides* seedlings, indicating that under drought stress, *E. ulmoides* seedlings enrich specific rhizosphere microbial taxa to cope with drought.

Based on the taxonomic data annotated by amplicon sequencing, we further explored the phylogenetic relationships of the enriched taxa in the rhizosphere of *E. ulmoides* under drought stress (Fig. 2B). The results showed that, with the exception of *Tilletia* and *Paxillus*, the enriched taxa all belonged to the order Sordariomycetes, including *Beauveria, Metarhizium, Moelleriella,* and *Ophiostoma*. These data indicate that drought stress affects the structure of the rhizosphere mycobiota and significantly enriches the microbial taxa of Sordariomycetes, which may have a positive impact on *E. ulmoides* adaptation to drought stress.

### Drought stress alters the co-occurrence patterns of the *E. ulmoides* mycobiota and the composition of the core fungal taxa

To further explore the co-occurrence patterns of the *E. ulmoides* rhizosphere mycobiota under different drought treatments, as well as the composition of the core fungal taxonomic taxa. The results showed that the treatment group drought 5 had the highest number of nodes and edges (node = 71; edge = 178), while drought 2 and drought 4 treatment groups had the fewest nodes (node = 31), with drought 2 having the fewest edges (edge = 61). Additionally, the control group had the lowest average degree (average degree = 3.707), whereas the drought 4 group had the highest average degree (average degree = 7.806). The control group had the highest modularity (modularity = 0.851), while drought 4 had the lowest modularity (modularity = 0.126) (Table 1). Importantly, the average path length and network diameter of the mid-drought treatments (i.e., drought 2, drought 3, and drought 4) were all shorter than those of the control group, drought 1, and drought 5. However, the average clustering coefficient of the mid-drought treatments was higher compared to the other groups, indicating that the mid-drought treatments have higher network connectivity and a more tightly knit network structure, exhibiting small-world” properties. Overall, drought stress led to changes in the interactive network of the *E. ulmoides* rhizosphere mycobiota, with stronger interactions during the mid-drought period.

**TABLE 1:** Co-occurrence network parameters of *E. ulmoides* rhizosphere microbes under drought stress

Network parameter	Node	Edge	Average degree	Modularity	Average clustering coefficient	Average path length	Network diameter
Control	41	76	3.707	0.851	0.517	4.784	12
Drought 1	44	99	4.500	0.610	0.500	4.621	11
Drought 2	31	61	3.935	0.418	0.587	2.617	7
Drought 3	40	106	5.30	0.554	0.663	3.082	7
Drought 4	31	121	7.806	0.126	0.768	2.179	7
Drought 5	71	178	5.014	0.785	0.557	5.790	15

**Fig 3:**
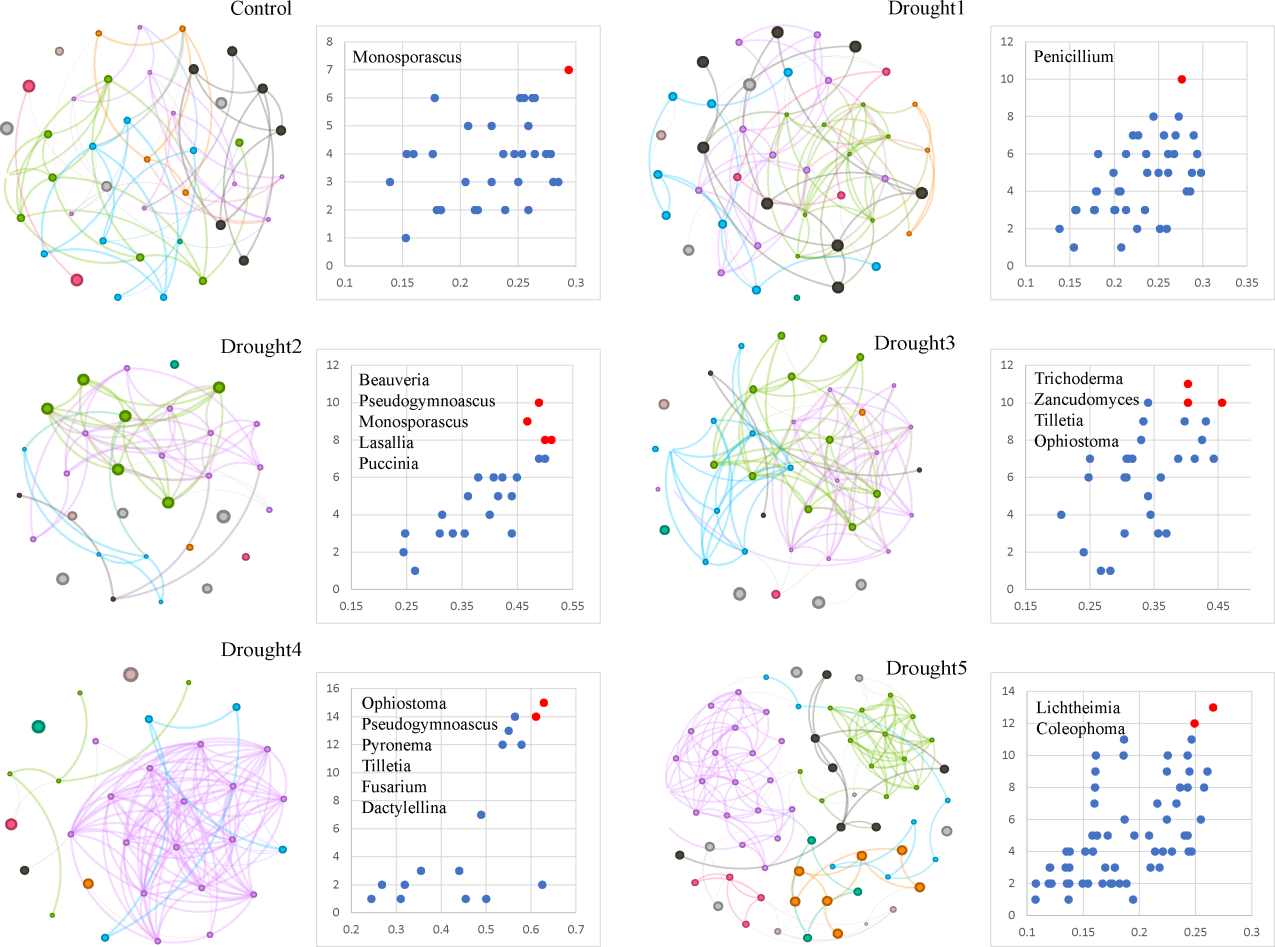
Co-occurrence network of *E. ulmoides* rhizosphere microbes under drought stress and core microbial taxa.

Utilizing network topological parameters, including degree and closeness centrality, we identified the composition of core fungal taxa at different drought stages (Fig. 3). The results revealed notable differences in core microbial taxa at the genus level across various drought periods. In the control group, *Monosporascus* served as a core mycobiota group. At the onset of drought (i.e., drought 1), *Penicillium* became the core microbial group. As the drought intensified (i.e., during the mid-drought period: drought 2–drought 4), the core microbial taxa diversified, including genera such as *Beauveria, Monosporascus, Lasallia, Puccinia, Pseudogymnoascus, Trichoderma, Zancudomyces, Tilletia,* and *Ophiostoma*, which exhibited higher network connectivity and influence. Notably, in the late drought stage (i.e., drought 5), *Lichtheimia* and *Coleophoma* emerged as core microbial taxa. These findings demonstrate dynamic changes in the interactive network of *E. ulmoides* rhizosphere microbes and core taxa under drought stress.

### The impact of rhizosphere fungal communities on the physiology of *E. ulmoides* leaves under drought stress

**Fig 4:**
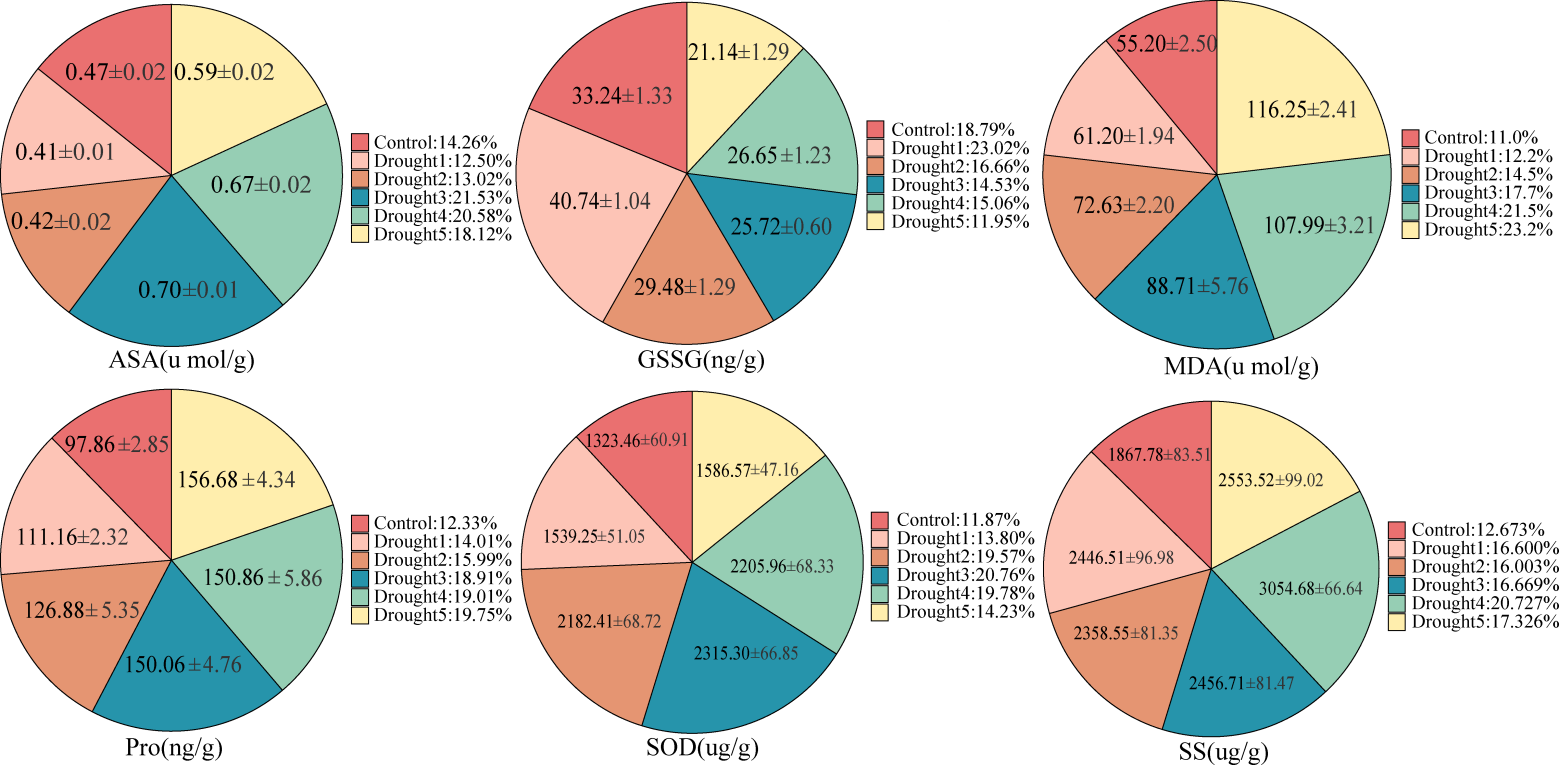
Changes in the levels of ascorbic acid, glutathione oxidized, malondialdehyde, proline, superoxide dismutase, and soluble sugar in *E. ulmoides* seedlings under drought stress.

To determine the relationship between *E. ulmoides* rhizosphere microbes and plant physiological indicators under drought stress, we measured the levels of AsA, GSSG, MDA, Pro, SOD, and SS in *E. ulmoides* leaf tissues (Fig. 4). The results showed that the AsA content initially increased and then decreased with increasing drought stress, peaking at 21.53% during drought 3. In contrast, GSSG content decreased with increasing drought intensity. In addition, MDA and Pro expression were upregulated with increasing drought intensity, with both peaking in the late drought period (i.e., drought 5). The levels of SOD and SS first increased before decreasing with the increasing drought stress, peaking at drought 3 and drought 4, respectively.

**Fig 5:**
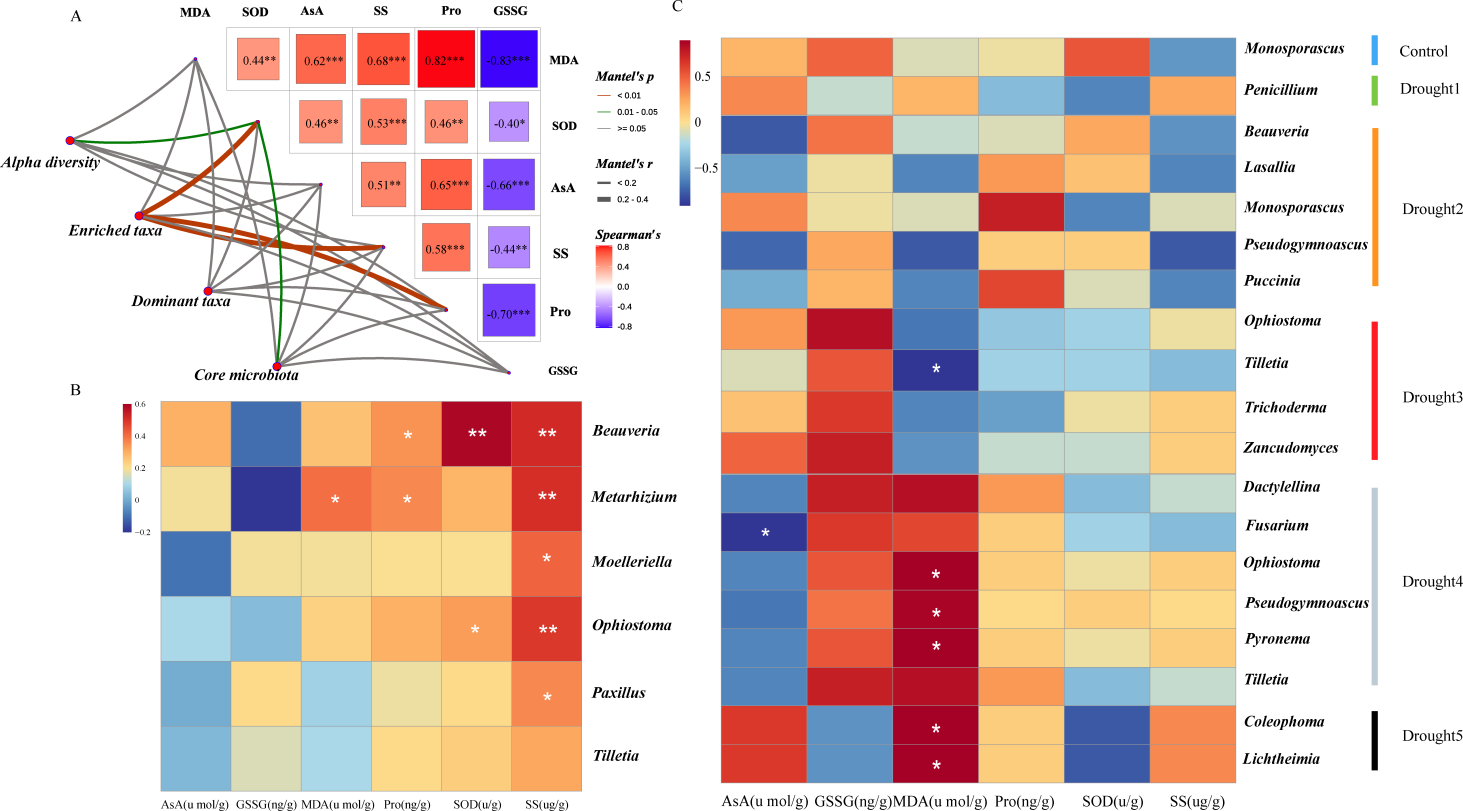
The relationship between *E. ulmoides* rhizosphere microbes and plant physiological indicators under drought stress. (**A**) Mantel test analysis of the relationships between dominant microbial taxa, enriched taxa, alpha diversity, core microbial taxa, and plant physiological indicators, including ascorbic acid, glutathione oxidized, malondialdehyde, proline, superoxide dismutase, and soluble sugar. (**B**) Enriched microbial taxa. (**C**) Heatmap analysis of the correlation between core microbial taxa and plant functional traits. Significance levels were *P < 0.05, **P < 0.01, ***P < 0.001.

Under drought stress, the enriched taxa in the *E. ulmoides* rhizosphere were significantly positively correlated with SOD, SS, and Pro. Furthermore, alpha diversity and the core microbial taxa exhibited a significant positive correlation with SOD (Fig. 5A). However, the dominant taxa showed no significant correlation with the six physiological indicators of plant leaf tissue. Overall, the enriched taxa had a closer relationship with the enzymatic activity in the *E. ulmoides* leaf tissue, and SOD showed a significant positive correlation with both the enriched taxa, alpha diversity, and the core microbial taxa.

Further analyses found that the enriched taxa *Beauveria, Metarhizium, Moelleriella,* and *Ophiostoma* had a more significant impact on SOD and SS (Fig. 5B). Interestingly, all four enriched taxa belong to the Sordariomycetes order. In contrast, the core microbial taxa from drought 4 and drought 5 showed a stronger positive correlation with MDA (Fig. 5C). Taken together, these data show that the enriched taxa have a greater influence on SS, while the core taxa in the later stages of drought have a greater impact on MDA (Fig. 5B and C). These results reflect a significant association between the *E. ulmoides* rhizosphere mycobiota and closely stress-related enzyme activity and mediators within the plant, showing that these microbial taxa may influence plant adaptability and tolerance to drought stress by participating in the regulation of physiological processes.

### The relationship between enriched taxa, core fungal taxa, and differentially expressed root metabolites

**Fig 6:**
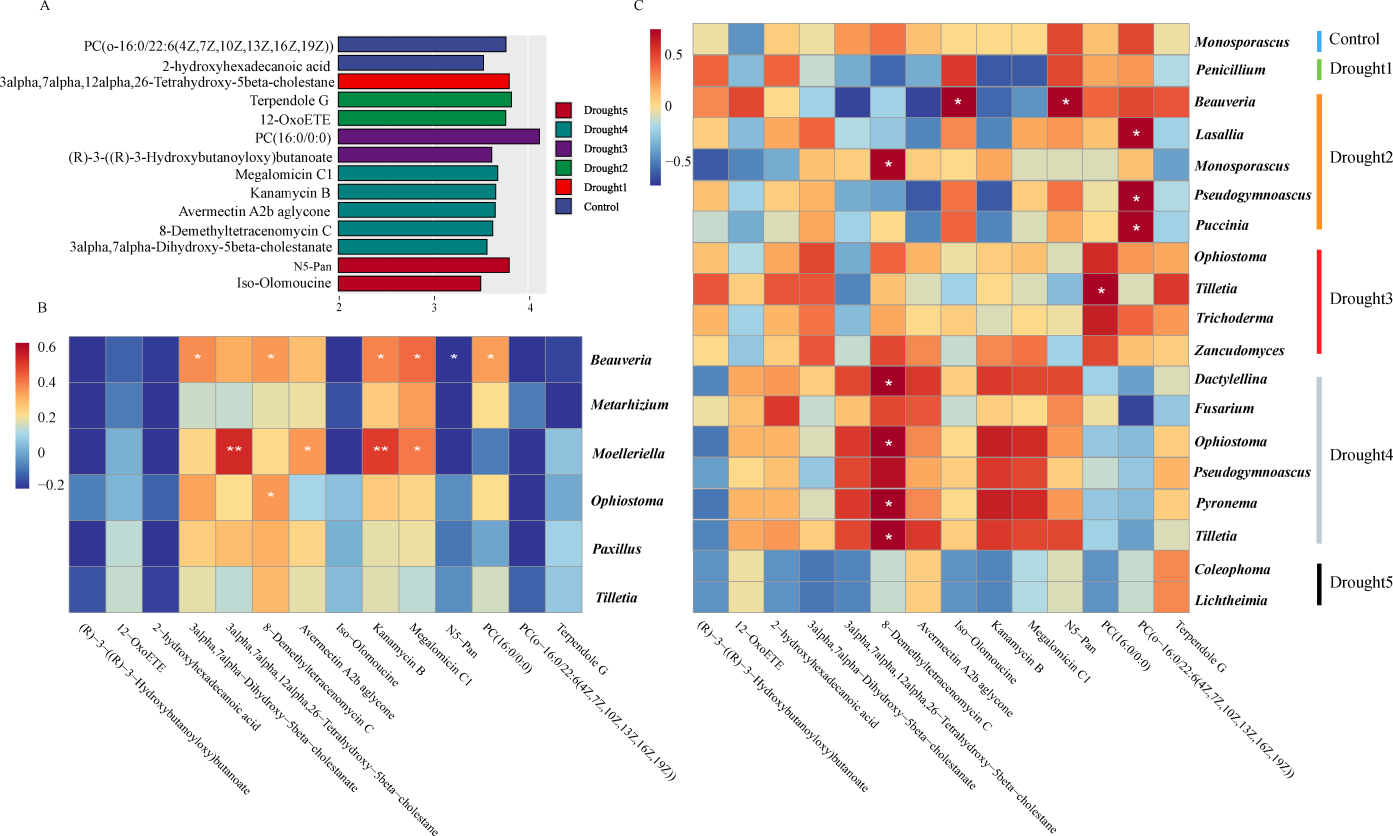
The relationship between enriched taxa, core microbial taxa, and differential rhizosphere metabolites under drought stress. (**A**) Bar chart of differential metabolites based on Lefse analysis, LDA = 3.5. (**B**) Correlation heatmap analysis between enriched taxa and (**C**) core microbial taxa with differential *E. ulmoides* rhizosphere metabolites. Significance levels were *P < 0.05, **P < 0.01.

To further explore the role of enriched taxa and core microbial communities in the growth of *E. ulmoides* seedlings under drought stress, we performed high-throughput sequencing of the rhizosphere metabolites of *E. ulmoides*. Using linear discriminant analysis effect size (LEfSe) analysis, 14 differential metabolites were identified, namely phosphatidylcholines (PC) (o-16:0/22:6(4Z,7Z,10Z,13Z,16Z,19Z)), 2-hydroxyhexadecanoic acid, 12-OxoETE, 3alpha,7alpha,12alpha,26-tetrahydroxy-5beta-cholestane, N5-Pan, (R)-3-((R)-3-hydroxybutanoyloxy)butanoate, terpendole G, 8-demethyltetracenomycin C, 3alpha,7alpha-Dihydroxy-5beta-cholestanate, PC (16:0/0:0), iso-olomoucine, kanamycin B, avermectin A2b aglycone, and megalomicin C1 (Fig. 6A). Furthermore, the dynamic changes of the 14 differential metabolites during various drought periods were analyzed, revealing that the content of 8-demethyltetracenomycin C in the Control group was the lowest and showed an increasing trend with the enhancement of drought stress. Additionally, the content of PC (16:0/0:0) exhibited a trend of increasing initially and then decreasing, reaching its peak in drought 3 (Fig. S2).

Spearman correlation analysis revealed significant relationships among enriched taxa. *Beauveria* showed a significant negative correlation with N5-Pan and a positive correlation with 8-demethyltetracenomycin C, 3alpha,7alpha-dihydroxy-5beta-cholestanate, PC (16:0/0:0), kanamycin B, and megalomicin C1. Additionally, 3alpha,7alpha,12alpha,26-tetrahydroxy-5beta-cholestane and kanamycin B exhibited a highly significant positive correlation with *Moelleriella* (Fig. 6B). Furthermore, 8-demethyltetracenomycin C was significantly correlated with the core taxa of *Ophiostoma, Pyronema, Tilletia, Dactylellina,* and *Monosporascus* (Fig. 6C). These results indicate that specific metabolites are intricately linked with key microbial taxa, potentially playing important roles in plant physiological processes.

## DISCUSSION

### Drought stress-induced changes in the composition of rhizosphere fungal communities and enrichment of the *Sordariomycetes* group

In this study, we investigated the impact of drought stress on the diversity and composition of the rhizosphere microbial community of *E. ulmoides* seedlings. The results showed that in the mid- and late stages of drought (i.e., drought 4 and drought 5 treatment groups), the relative abundance of the phylum Ascomycota was significantly higher than in other groups (Fig. 1C). Previous studies have shown that soil microbial communities that have experienced drought interact with their hosts to enhance host adaptability under drought stress (32). In the natural world, the close symbiotic relationships between fungi and living plants, whether acting as mycorrhizal partners or forming parasitic relationships, are widely recognized for their significant role in shaping the structure of plant communities (16, 33, 34). There is also evidence to suggest that adverse conditions induce changes in the rhizosphere mycobiota due to the combined effects of biotic and abiotic stressors and the host plant (35). Under drought stress, both the host plant and the drought stress can cause changes in the rhizosphere mycobiota (36). Our findings not only corroborate previous research on significant changes in rhizosphere mycobiota under drought stress but also extend the understanding of these dynamics. This insight provides a foundation for future research on targeted microbial interventions to enhance plant drought resilience. For instance, research by Pan et al. has shown that drought stress can lead to the reassembly of the rhizosphere mycobiota, and these fungal communities enhance stress-induced adaptation in plants when challenged by drought. Furthermore, *Chaetomium* fungi have also been shown to improve drought resistance in plants (18). Similarly, under drought conditions, fungal communities associated with rice root systems undergo changes, leading to increased species diversity within the rice roots, which also indicates an increase in the relative abundance of Pezizomycotina (a subphylum of Ascomycota) under drought conditions (37). In agreement with previous findings, our data show that drought stress significantly affects the diversity and composition of the *E. ulmoides* seedling rhizosphere mycobiota, suggesting that changes in the rhizosphere mycobiota under drought conditions may be closely related to plant adaptability in response to drought stress.

Further analyses revealed that Sordariomycetes fungi were considerably enriched in the rhizosphere of *E. ulmoides* under drought stress, including *Beauveria, Metarhizium, Moelleriella,* and *Ophiostoma* (Fig. 2A). Notably, *Beauveria* fungi are recognized as entomopathogenic fungi and as endophytic symbionts of plants. These fungi can colonize a variety of plant species, protecting them from insect pests and promoting their growth and nutritional value (38). Inoculation with *Beauveria bassiana* has been shown to improve the growth rate of common beans and lettuce, along with physiological and antioxidant activity (39, 40). For example, research by Ferus et al. demonstrated that *B. bassiana* can increase drought resistance in *Quercus rubra* L. seedlings (19). Similarly, *Metarhizium*, a genus of entomopathogenic fungi commonly inhabiting the rhizosphere, is widespread in the soil and can provide benefits to a variety of host plants. These benefits include resistance to saline stress, increased plant biomass and growth (41), and stimulation of root growth (42). Compared with the previously mentioned fungi, there are relatively few studies on *Ophiostoma* under plant drought stress. Fortunately, studies have indicated that *Ophiostoma* spp. are among the endophytic fungi of *Ulmus minor* Mill. and are considered beneficial symbionts against stress. They can activate a plant’s antioxidant system in conjunction with the action of other endophytes (43, 44). The antioxidant system in plants plays a positive role in improving drought resistance. Therefore, we speculate that under drought stress, the rhizosphere of *E. ulmoides* may enrich *Sordariomycetes* fungi as a strategy to cope with drought stress, which also provides a theoretical reference for the development of future fungal inoculants (45).

### Under drought stress, the rhizosphere of *E. ulmoides* may resist drought by changing core fungal taxa

Co-occurrence networks can reveal the interconnections between different microbial species and identify core fungal taxa that are critical for maintaining ecosystem functions (46). In this study, the co-occurrence network of *E. ulmoides* rhizosphere microbes during the mid-stages of drought (i.e., drought 2, drought 3, and drought 4) exhibited higher network connectivity and a more tightly knit structure compared to the control group as well as the early and late stages of drought (Table 1; Fig. 3). This indicates that the interactions between fungi in the rhizosphere of *E. ulmoides* are more intense during the mid-stages of drought, exhibiting the properties of a “small-world” network. A previous study has shown that drought conditions reduce the correlation between bacteria and fungi in drought-resistant wheat varieties, further leading to decreased network complexity and severely limited interactions between microbes. Moreover, fungi can resist drought damage by enhancing the positive correlation between species (47). Yu et al. investigated the co-occurrence networks of soil bacteria and fungi in the XueYue Mountain area and found that fungi are superior to bacteria in maintaining the microbial symbiotic network (46). Similarly, work by Benidire et al. indicated that an increase in the complexity of the root microbiome interaction network in broad bean (*Vicia faba*) helps to enhance host tolerance to salt stress (48). In this study, the network connectivity of the *E. ulmoides* rhizosphere microbiota decreased in the later stages of drought, indicating that drought stress may significantly affect patterns of microbial interactions within the microbial co-occurrence network, thereby impacting drought resistance in plants.

Additionally, in this study we also show that with increasing drought stress, there were significant differences in the core microbial taxa in the rhizosphere of *E. ulmoides* at different drought intensities (Fig. 3). The core microbiome has been proven to play an important role in host growth and performance, making it a suitable target for studying plant phenotypes or performance mediated by the microbiome (49–51). Importantly, core microbes such as *Trichoderma* and *Penicillium* have previously been shown to play a role in drought resistance in plants. For instance, *Trichoderma* species help corn and tobacco crops to cope with drought stress by increasing antioxidant activity (52, 53). Similarly, *Penicillium* species of fungi also upregulate antioxidant enzyme activity in rice plants in response to drought, accumulate SS’ and Pro, and increase root length to combat drought (54). Furthermore, research by Shen et al. has indicated that there are differences in the core microbial communities of banana rhizospheres under various stress conditions (55). Studies by Luo et al. have also demonstrated that heavy metal stress is a driving factor in the changes and re-organization of plant rhizosphere core microbial communities (56). Among the core microbial communities, some members, such as key taxonomic taxa, can shape the community composition through strong biological interactions with the host or other co-occurring microbial species (57). These findings highlight the crucial role of core microbes in facilitating plant adaptation to environmental stress. Similar to the enriched taxa , core microbes are also promising candidates for the development of future microbial inoculants. As mentioned by Li et al., candidate microbes can be identified through the analysis of high-throughput sequencing data to construct synthetic microbial communities. Following validation and optimization, these communities can be further developed into composite inoculants tailored for applications in agriculture and ecological settings (45).

### The rhizosphere fungi of *E. ulmoides* may contribute to drought tolerance by influencing host plant physiology and root metabolites

By measuring key physiological indicators in *E. ulmoides* leaves, we explored the association between drought stress and the rhizosphere fungi of *E. ulmoides*. From this, we identified a series of trends that changed with drought stress. As drought stress intensified, MDA, Pro, and SS in *E. ulmoides* leaves generally showed an increasing trend (Fig. 4). Concurrent with these changes, we also found that SOD activity and AsA content first increased and then decreased. Multiple studies have shown that increasing levels of drought stress lead to a gradual increase in MDA and SS levels (58, 59). Research by Zhang et al. showed that under drought stress, the expression of SS’ and Pro in *Santalum album* leaves significantly increased, and MDA accumulated in the plant (60). Importantly, these data also support our findings. Furthermore, within a certain range, the activity and expression of SOD also increased with stress. However, at higher levels of stress, SOD expression has been shown to decrease (59). Furthermore, under significant drought stress, SOD activity in rice seedlings rapidly decreases after exceeding the tolerance limit (61), which is consistent with our data showing that SOD content first increases followed by a decrease. Under drought stress, plants typically accumulate more reactive oxygen species (ROS), which usually leads to an increase in the levels of oxidized glutathione. However, in our study, we observed a decrease in GSSG levels. In conjunction with the review of recycling of GSSG by glutathione reductase (GR) by Dorion et al., this may be due to the enhanced activity of GR under drought stress (62), which converts GSSG back to reduced glutathione (63, 64).

In this study, we also found that MDA content showed a significant positive correlation with the core microbial taxa in the later stages of drought, whereas the correlation with SS was stronger in the enriched taxa (Fig. 5). Specifically, the enriched taxa, including *Beauveria*, *Metarhizium*, *Moelleriella*, and *Ophiostoma*, had a more notable impact on SS. Interestingly, phylogenetic analyses revealed that these groups all belong to the Sordariomycetes class. Sordariomycetes fungi have been widely reported in the field of plant drought resistance and have been shown to improve plants’ tolerance to drought stress through various mechanisms, thereby improving plant survival and growth. These mechanisms include enhancing the plant’s water absorption capacity, strengthening antioxidant activity, and promoting plant root growth (65–67). These results suggest that core microbial taxa and enriched taxa may synergistically enhance the drought resistance in *E. ulmoides* seedlings by affecting different physiological indicators.

There is an increasing body of evidence to show that the indirect effects produced by root exudates can offset the direct impacts of drought on the rhizosphere microbial community (68). However, studies on the correlation between endophytic metabolites and rhizosphere fungal communities remain scarce. To delve into the role of enriched taxa and core microbial communities on the growth of *E. ulmoides* seedlings under drought stress, we conducted a non-targeted metabolomic sequencing of the endophytic metabolites in *E. ulmoides* roots. Through LEfSe analysis, we identified 14 differentially expressed metabolites that may be associated with plant drought responses and their interactions with fungal communities (Fig. 6A). Among these metabolites, 3alpha,7alpha,12alpha,26-tetrahydroxy-5beta-cholestane and 3alpha,7alpha-dihydroxy-5beta-cholestanate are steroidal compounds. Previous research has suggested that enhancing the biosynthesis of sterols can significantly improve plant drought tolerance and disease resistance (69). Similarly, PC (16:0/0:0) and PC (o-16:0/22:6(4Z,7Z,10Z,13Z,16Z,19Z)) are specific phospholipids that belong to the class of phosphatidylcholines. Under drought conditions, the fluidity of plant cell membranes is reduced, which can impair cellular function (70). Phosphatidylcholines, such as PC (16:0/0:0) and PC (o-16:0/22:6), are crucial for maintaining the fluidity and integrity of cell membranes and can help maintain membrane integrity under such stress conditions (71). Furthermore, based on Spearman correlation analysis, 8-demethyltetracenomycin C showed significant correlations with core communities, including *Ophiostoma*, *Pyronema*, *Tilletia*, *Dactylellina*, and *Monosporascus*, indicating that these metabolites may play an important role in plant-microbe interactions. Notably, 8-demethyltetracenomycin C is a tetracycline-class compound, belonging to the polyketide class of natural products. It has been shown to possess antioxidant properties, which enable it to scavenge ROS and thereby mitigate oxidative stress (72). Therefore, 8-demethyltetracenomycin C may serve as an antioxidant for plants during periods of drought. Moreover, the *Beauveria* genus within the core microbial community showed a significant positive correlation with N5-Pan (a type of polyamine) (Fig. 6). These results suggest that the structure and function of the rhizosphere microbial community may influence plant drought resistance by affecting the synthesis and regulation of plant metabolites. For instance, the role of polyamines in plant drought and salt stress resistance is considered an emerging area of research interest because of their involvement in protecting plants against drought and soil salinization stress events, maintaining osmotic balance in plant cells under drought stress, and alleviating oxidative damage (73). Therefore, we hypothesize that under drought stress, the core mycobiota in the rhizosphere of *E. ulmoides* may help the plant to cope with drought by promoting host production of specific metabolites. Building upon our current correlation analysis of fungal communities and metabolites, future research should employ single-factor controlled experiments to further validate the findings. Specifically, experimental designs could include inoculating microorganisms to observe subsequent changes in metabolite profiles or applying specific metabolites to monitor shifts in microbial community composition, which could help elucidate the causal relationships between metabolites and fungal taxa.

## Data Availability

All genome data have been uploaded to NCBI under BioProject accession number PRJNA1151941.

## SUPPLEMENTAL MATERIAL

Supplemental File spectrum.00847-25-s0001.docx Supplemental figures and tables.

ASM does not own the copyrights to Supplemental Material that may be linked to, or accessed through, an article. The authors have granted ASM a non-exclusive, world-wide license to publish the Supplemental Material files. Please contact the corresponding author directly for reuse.
