# Supplementary material for: Drought-driven shifts in Eucommia ulmoides rhizosphere mycobiota and metabolites mediate host tolerance
Source: Microbiol Spectr. 2025 Jul 11;13(8):e00847-25. doi: 10.1128/spectrum.00847-25 (PMC12323333; doi:10.1128/spectrum.00847-25)
Supplement: Supplemental File — Supplemental figures and tables. [file spectrum.00847-25-s0001.docx]

**Table S1.** Basic soil nutrients.

| Ingredient | Quantity |
| --- | --- |
| Organic matter content (g/kg) | 6.6 ± 1.3 |
| Available phosphorus (mg/kg) | 51.4 ± 3.7 |
| Total nitrogen (mg/kg) | 799.2 ± 13.1 |
| Available nitrogen (mg/kg) | 847.3 ± 11.1 |
| Available potassium (mg/kg) | 358.6 ± 7.7 |
| pH | 0.71 ± 0.04 |

**Table S2.** Soil water content in each treatment group.

| Group | Soil moisture content |
| --- | --- |
| Control | 32.63 ± 0.61 |
| Drought 1 | 21.83 ± 0.51 |
| Drought 2 | 12.17±0.87 |
| Drought 3 | 9.48 ± 0.71 |
| Drought 4 | 6.49 ± 0.21 |
| Drought 5 | 5.67±0.17 |

**
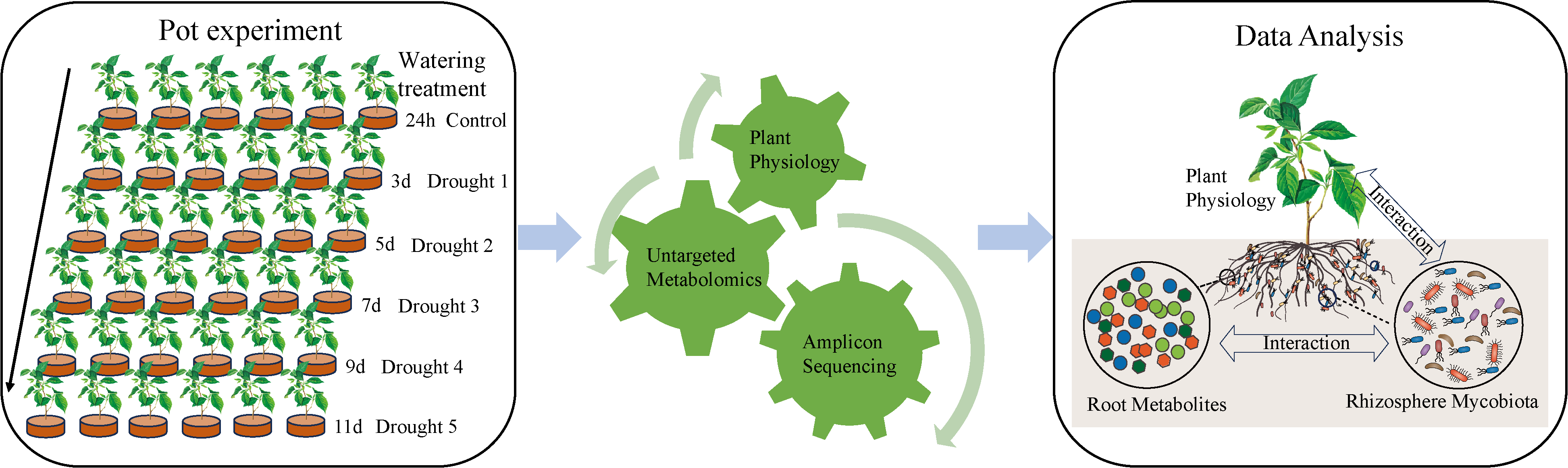
**

**Figure S1.** Schematic diagram of experimental design, data sources, and data analysis.

**
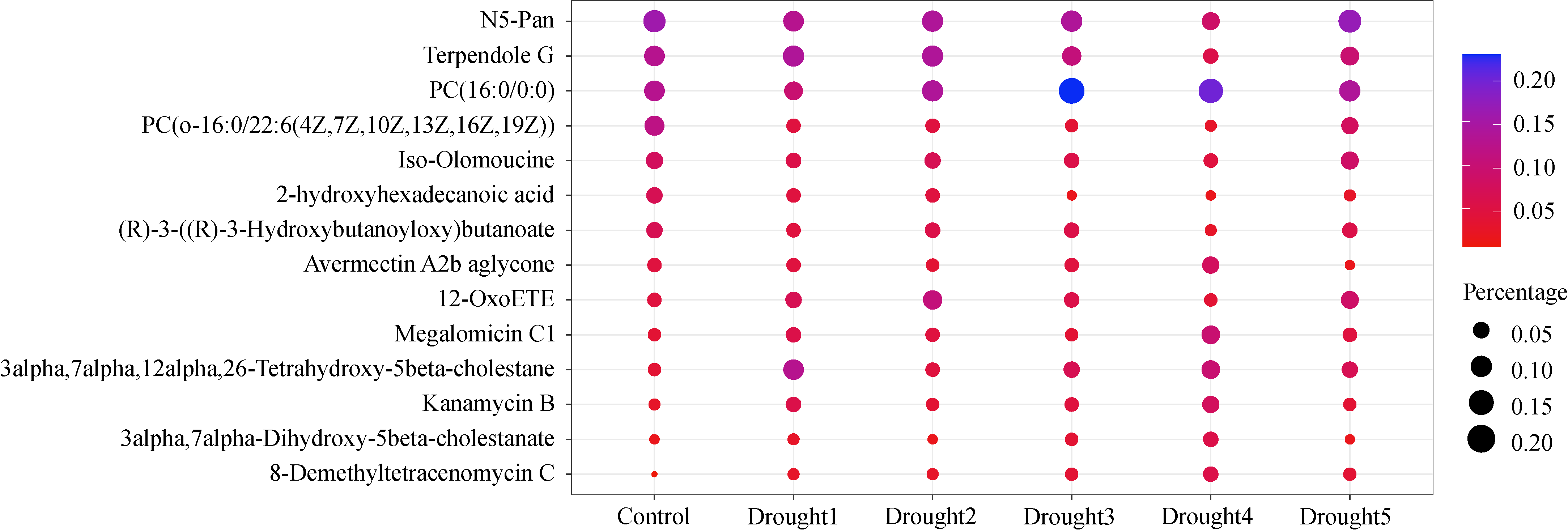
**

**Figure S2.** Bubble chart showing the dynamic changes of 14 differential metabolites.

The steps for extracting rhizosphere soil DNA using the CTAB method are as follows: Briefly, 500 mg of the sample was placed into a 2 mL centrifuge tube and thoroughly ground in liquid nitrogen. Subsequently, 1 mL of pre-warmed (65 °C) CTAB extraction buffer, 2% (v/v) β-mercaptoethanol, and 10% (w/v) lysozyme were added to the sample tube, vortexed to mix, and then incubated at 65 °C for 40 – 60 minutes with mixing 2 – 3 times to ensure complete lysis. After incubation, the samples were cooled to room temperature and centrifuged at 12,000 rpm for 5 minutes, and 900 μL of the supernatant was transferred to a new 2 mL sterile centrifuge tube. An equal volume of chloroform/isoamyl alcohol (24:1) was added, mixed thoroughly by inverting, and then centrifuged at 12,000 rpm for 20 minutes to pellet the DNA. Subsequently, 700 μL of the supernatant was transferred to a new 2 mL sterile centrifuge tube, and the previous step was repeated to further purify the DNA. Next, 450 μL of the supernatant was transferred to a 1.5 mL sterile centrifuge tube, and isopropanol (two thirds of the volume of the supernatant) and 3 M sodium acetate (one tenth of the volume) were added, mixed well, and then stored at −20 °C for 1 hour to precipitate the DNA. After precipitation, the samples were centrifuged at 12,000 rpm for 10 minutes, the supernatant was discarded, and any excess liquid was removed by brief centrifugation and aspiration. Next, 1 mL of 75% (v/v) ethanol solution was added, the pellet was gently resuspended by flicking, and after centrifugation for 5 minutes, the supernatant was discarded again. Finally, the samples were briefly centrifuged, any remaining liquid was discarded, and the tubes were left open to air-dry at room temperature for 3 – 5 minutes until the DNA pellet appeared translucent. Sufficient ultra-pure water (at least 20 μL) containing 10 ng/μL of RNaseA was added, the volume was adjusted based on the size of the pellet to dissolve the DNA precipitate, and the samples were incubated in a 37 °C water bath for 1 hour. The dissolved DNA was subsequently stored at −20 °C for future use.
